# A Comparison of Mental Health in a Sample of Older Women Before and During the COVID-19 Pandemic

**DOI:** 10.1093/geroni/igab046.2678

**Published:** 2021-12-17

**Authors:** Victoria Marshall, Robina Sandhu, Kathryn Kanzler, Sara Espinoza, Pamela Keel, Andrea LaCroix, Nicolas Musi, Lisa Kilpela

**Affiliations:** 1 University of Texas Health Science Center San Antonio, San Antonio, Texas, United States; 2 University of Texas Health Science Center, San Antonio, Texas, United States; 3 UT Health San Antonio, San Antonio, Texas, United States; 4 Florida State University, Tallahassee, Florida, United States; 5 University of California at San Diego, San Diego, California, United States

## Abstract

The COVID-19 pandemic has substantially impacted lives globally. Due to age-related risks, the older adult population has uniquely experienced negative changes caused by the pandemic. Research has also shown that the pandemic has disproportionately affected women. Therefore, it is important to understand how the mental health of older women has been impacted during this global crisis. This study aims to examine the differences in mental health indices in a sample of older women before versus during the COVID-19 pandemic. To date, participants include 201 women (aged 60-94) who completed an online survey of self-report measures assessing depression, anxiety, alcohol use, binge eating, positive affect, and emotional quality of life (QOL). We conducted one-way ANOVAs to compare each mental health construct in two samples of older women collected pre- and peri-pandemic. Results indicated that the peri-pandemic group reported significantly higher anxiety (F = 5.25, p = .02), with a trend for more role limitations due to emotional problems (F = 2.79, p = .09), than the pre-pandemic group. No significant differences emerged for levels of depression, alcohol consumption, binge eating, positive affect, or emotional wellbeing between groups. These findings point to the psychological resilience of older adults in the face of extreme adverse events, including this global crisis. Older women, while impacted differently during the COVID-19 pandemic, reported minimal exacerbations of mental health problems compared to older women pre-pandemic. Efforts to identify moderators that may either attenuate or promote further psychological resilience among older adults is warranted.

